# Pediatric Measles and Visceral Leishmaniasis Co-infection: Diagnostic Challenges and Insights Into Pathophysiology

**DOI:** 10.7759/cureus.112957

**Published:** 2026-07-19

**Authors:** Kalliopi Straka, Eleni Christakou

**Affiliations:** 1 Pediatric Intensive Care Unit, National and Kapodistrian University of Athens, Athens, GRC; 2 Pediatric Intensive Care Unit, Aghia Sophia Children's Hospital, Athens, GRC

**Keywords:** co-infection, immune amnesia, measles, pediatric infection, picu, visceral leishmaniasis

## Abstract

Measles remains a major cause of childhood morbidity worldwide despite the availability of an effective vaccine. Beyond its acute clinical manifestations, measles induces profound but transient immune dysfunction, predisposing affected children to secondary and opportunistic infections. Visceral leishmaniasis (VL) is a systemic protozoal infection caused by *Leishmania* species and is characterized by prolonged fever, hepatosplenomegaly, pancytopenia, and high mortality if left untreated. The coexistence of severe measles and VL poses a significant diagnostic challenge because of their overlapping clinical and laboratory features. We report the case of a previously healthy three-year-old boy admitted to the pediatric intensive care unit with severe PCR-confirmed measles complicated by acute respiratory failure and hemodynamic instability requiring invasive mechanical ventilation and vasoactive support. Persistent fever, progressive hepatosplenomegaly, and cytopenias despite initial intensive management prompted further investigation, which demonstrated positive rK39 antigen testing and elevated anti-*Leishmania* antibody titers, establishing the diagnosis of VL. The patient was successfully treated with liposomal amphotericin B, vitamin A supplementation, IV immunoglobulin, and supportive intensive care, resulting in complete clinical recovery without sequelae. To the best of our knowledge, no pediatric case of measles-VL co-infection has been documented previously. This case highlights the diagnostic complexity of persistent systemic inflammation and cytopenias in children with severe measles and illustrates the importance of considering endemic opportunistic infections when the clinical course is atypical or prolonged. The favorable outcome emphasizes the value of early recognition, timely pathogen-directed therapy, and multidisciplinary management, while reinforcing the critical role of measles vaccination in preventing severe disease and its infectious complications.

## Introduction

Measles remains one of the most contagious viral infections known to humans [1]. Despite the availability of a safe and highly effective vaccine, measles continues to pose a substantial global health burden. In 2022, the World Health Organization reported more than 200,000 measles-related deaths worldwide, predominantly affecting children under five years old [2]. Recent outbreaks across Europe highlight persistent gaps in vaccination coverage and increasing vaccine hesitancy, leaving children vulnerable to acute infection and its serious complications.

Beyond its acute manifestations, measles is increasingly recognized as a cause of profound and prolonged immune dysfunction. The measles virus preferentially infects immune cells expressing CD150, also known as signaling lymphocytic activation molecule (SLAM), including memory B cells, T lymphocytes, and dendritic cells, resulting in widespread depletion of immunological memory [3]. This phenomenon, termed "immune amnesia," refers to the partial loss of pre-existing immune memory against previously encountered pathogens and may persist for months to years following measles infection [4,5]. As a result, affected children become more susceptible to secondary bacterial, viral, and, rarely, opportunistic parasitic infections.

Secondary bacterial infections, such as pneumonia and otitis media, are well-described complications following measles infection [6]. In contrast, parasitic co-infections remain exceedingly rare. Visceral leishmaniasis (VL), caused by protozoa of the *Leishmania donovani* complex, is a systemic parasitic disease characterized by prolonged fever, hepatosplenomegaly, and pancytopenia [7]. Effective control of infection relies on intact Th1-mediated immunity, particularly interferon-γ (IFN-γ)-driven macrophage activation [8]. Measles-induced immune suppression is characterized by a shift from Th1- to Th2-mediated immune responses, accompanied by reduced IFN-γ production and impaired macrophage activation. Because intracellular killing of *Leishmania* depends largely on IFN-γ-activated macrophages, this immunological shift may facilitate parasite persistence or the progression of infection [8].

Pediatric VL carries mortality rates approaching 90% if untreated [7]. Greece is considered an area of sporadic VL transmission, predominantly due to *Leishmania infantum* [9]. Although Greece is considered a low-incidence country for measles following widespread vaccination, periodic outbreaks persist due to suboptimal vaccine coverage. At the same time, *Leishmania infantum* remains endemic in several Mediterranean regions, including Greece [2,9]. To the best of our knowledge, no pediatric case of measles-VL co-infection has been documented previously. We describe a critically ill toddler with severe measles complicated by VL and discuss the diagnostic challenges together with the potential immunopathological mechanisms underlying this rare association.

## Case presentation

A three-year-old boy was transferred by air ambulance to the pediatric intensive care unit (PICU) from a rural hospital, intubated and ventilated for acute respiratory failure and hemodynamic instability in the setting of an ongoing febrile illness.

He was the third child in the family, born at term by spontaneous vaginal delivery (birth weight 2860 g) following inadequate prenatal care and an unremarkable perinatal period. The parents were second-degree relatives. His vaccination history was unknown. Family history was notable for epilepsy in a maternal great-aunt. One year prior to his admission, the patient had been hospitalized for an episode of transient loss of consciousness with hypotonia and perioral cyanosis; neurological evaluation, including EEG, was unremarkable. He also underwent evaluation from a pediatric cardiologist, whose examination was also normal. He had since been receiving levetiracetam per os (30 mg/kg/day) without recurrence of events, and his growth and psychomotor development were appropriate for his age. A household contact had been diagnosed with laboratory-confirmed measles approximately two weeks prior to symptom onset. The family reported no history of travel or exposure to VL-endemic areas outside Greece.

The current illness began five days before admission to our PICU (Day -5) with fever and diarrhea. He was initially evaluated in the emergency department and diagnosed with acute otitis media, for which oral antibiotics were prescribed (Day -5). On Day -2, because of persistent fever, diarrhea, poor oral intake, and lethargy, he re-presented to the referring hospital. Given the history of household exposure to laboratory-confirmed measles approximately two weeks earlier along with his clinical deterioration, he was admitted to the pediatric ward for further evaluation. Upon admission, the patient was found to be critically ill with a high temperature of 39.8°C, heart rate of 165 beats/min, respiratory rate of 50 breaths/min, blood pressure of 70/40 mmHg, and oxygen saturation of 85% on room air. Physical examination revealed diffuse maculopapular rash, marked respiratory distress, and hepatosplenomegaly (palpable liver 4 cm and spleen 5 cm below the costal margin). Neurologically, he was drowsy but without any specific focal deficits. Shortly thereafter (Day -1), he developed altered mental status, severe respiratory distress with hypoxemia and metabolic acidosis (ABG: pH 7.09, pCO₂ 70 mmHg), and hemodynamic instability requiring urgent endotracheal intubation, mechanical ventilation, and inotropic support before transfer to our tertiary PICU. Following PICU admission (Day 0), measles infection was confirmed by nasopharyngeal PCR, while persistent fever, hepatosplenomegaly, and pancytopenia prompted further investigation. VL was diagnosed shortly thereafter following positive rK39 antigen testing and elevated anti-*Leishmania* antibody titers, allowing prompt initiation of treatment with liposomal amphotericin B.

At the same time, due to hemodynamic instability, he was initiated on inotropic support. His laboratory investigations demonstrated a marked inflammatory response and abnormal full blood count: CRP 170 mg/L (normal values <5 mg/L), procalcitonin 13.3 ng/mL (normal values <0.5 ng/mL), elevated WBC 12800/μL with 78% polymorphonuclear neutrophils and 19% lymphocytes, anemia (Hb 7.5 g/dL), thrombocytopenia (45 × 10⁹/L), hyponatremia (Na 125 mmol/L), and elevated D-dimers. Empiric IV antibiotics were escalated, and the patient was transferred by air ambulance to a tertiary hospital and admitted immediately to PICU for further management.

Upon admission, the patient was sedated with equal, reactive pupils and was hypotensive (BP: 88/37 mmHg) and tachycardic (HR: 110 beats/min), requiring combined inotropic and vasopressor support with dobutamine and norepinephrine infusion. Capillary refill time was 2 seconds, peripheral pulses were palpable, and urine output was adequate. Oxygen saturation was maintained at 99% under mechanical ventilation. Physical examination revealed decreased bilateral air entry with crackles and adequate chest expansion and hepatosplenomegaly (liver palpable 4 cm below the costal margin; spleen 5 cm with firm consistency). He was noted to have conjunctival hyperemia with eyelid edema, facial erythema, and right tympanic membrane rupture, findings consistent with acute otitis media. Laboratory findings demonstrated markedly elevated inflammatory markers (CRP 131 mg/L; procalcitonin peaking at 13.3 ng/mL, then 18.27 ng/mL), anemia, thrombocytopenia, mild hyponatremia, and hypoalbuminemia. Liver and renal function tests remained within normal limits.

His extended diagnostic workup included blood, respiratory, urine, and rectal cultures, multiplex PCR for sepsis pathogens, and respiratory samples for viral testing. Viral testing (nasopharyngeal swab PCR) was positive for measles, confirming active measles virus infection. Persistent fever, cytopenias, and hepatosplenomegaly prompted evaluation for parasitic infections. Serologic testing for *Leishmania* (rK39 antigen) was positive, and elevated anti-*Leishmania* antibody titers confirmed the diagnosis, while influenza antigen, SeptiFast panel, and blood cultures were negative. A chest X-ray (CXR) demonstrated diffuse interstitial infiltrates consistent with viral pneumonitis. Abdominal ultrasound confirmed hepatosplenomegaly. Repeated blood cultures obtained during the PICU stay also remained sterile, making persistent bacteremia unlikely. As expected, blood cultures were not useful for diagnosing VL, which was established by positive serological testing, the characteristic clinical presentation, and a subsequent response to antiparasitic therapy.

Given the combination of persistent fever, hepatosplenomegaly, and progressive cytopenias despite appropriate intensive care and broad-spectrum antimicrobial therapy, the differential diagnosis included hemophagocytic lymphohistiocytosis (HLH), hematological malignancy, and viral infections such as Epstein-Barr virus and cytomegalovirus. However, the positive rK39 antigen test, together with elevated anti-*Leishmania* antibody titers, the epidemiological setting, and the subsequent rapid clinical response to liposomal amphotericin B, strongly supported the diagnosis of VL.

Although secondary HLH was considered because of persistent fever, hepatosplenomegaly, cytopenias, and hyperferritinemia, the patient did not fulfill the diagnostic criteria, and his rapid clinical and laboratory improvement following targeted antiparasitic therapy argued against this diagnosis. Regarding the leishmaniasis diagnosis, bone marrow examination and molecular confirmation (PCR) were not performed because the combination of compatible clinical findings, positive serology, and rapid response to liposomal amphotericin B was considered sufficient to establish the diagnosis, thereby avoiding additional invasive procedures in a critically ill child.

The patient remained mechanically ventilated for six days, initially requiring high ventilator parameters to achieve adequate ventilation and oxygenation, with gradual improvement in arterial gas exchange. Following extubation, the patient developed acute post-extubation stridor and hypercapnia, which responded to IV dexamethasone, nebulized adrenaline, and salbutamol. Respiratory status subsequently stabilized, and the patient was discharged from the PICU breathing spontaneously on room air with oxygen saturation of 100%. Hemodynamic stability was gradually achieved, allowing discontinuation of vasoactive support by day six of hospitalization. Diuretic therapy with furosemide IV was administered transiently for fluid management. Cardiac function normalized, and the patient remained hemodynamically stable thereafter with normal ECG and echocardiogram findings. Neurologically, the patient remained sedated for six days. After discontinuation of sedation and extubation, the patient was awake and alert, with equal and reactive pupils, visual tracking, purposeful limb movements, and appropriate interaction. Antiepileptic therapy with levetiracetam was continued. Enteral feeding via a nasogastric tube was well tolerated, with a transition to oral intake prior to discharge.

**Figure 1:**
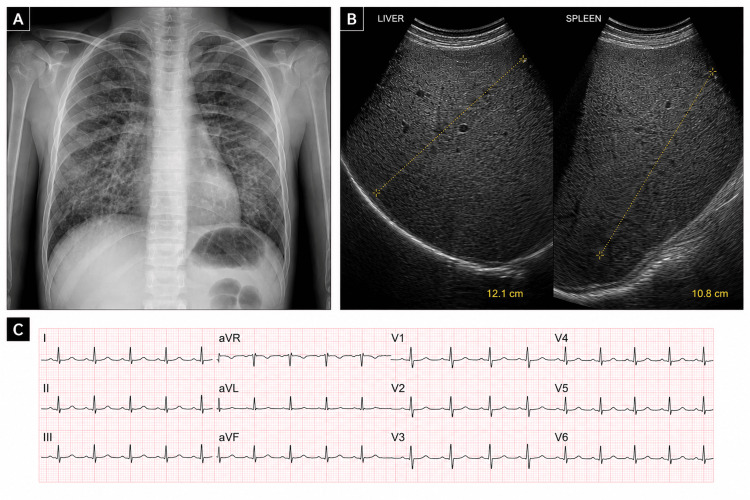
Radiological and cardiac findings in a three-year-old child with severe measles-VL co-infection (A) Chest radiograph demonstrating diffuse bilateral perihilar and interstitial pulmonary infiltrates, consistent with viral pneumonitis. (B) Abdominal ultrasound demonstrating hepatomegaly and splenomegaly without focal hepatic or splenic lesions. (C) Twelve-lead ECG demonstrating normal sinus rhythm with age-appropriate intervals and no conduction abnormalities. VL: visceral leishmaniasis, ECG: electrocardiogram

The patient's imaging (CXR, abdominal ultrasound, and ECG) are shown in Figure 1.

Following the pediatric infectious diseases specialist’s consultation, he received targeted therapy, including liposomal amphotericin B (total dose of 20 mg/kg) for VL, high-dose vitamin A supplementation, IV immunoglobulin (1 g/kg for two days) for measles-associated immunosuppression, and broad-spectrum antibiotics intravenously (cefotaxime and teicoplanin). Clinical improvement became evident within 72 hours of initiating antiparasitic therapy, with defervescence, rising blood counts, and gradual resolution of hepatosplenomegaly. Empiric antibiotics were discontinued after negative cultures and clinical stabilization. The patient also received albumin supplementation, packed red blood cells, and platelet transfusions for anemia and thrombocytopenia. Supportive care was continued with fluid and nutritional management. Inflammatory markers progressively declined (CRP from 131 to 9.6 mg/L; procalcitonin from a peak of 18.3 to 0.52 ng/mL) prior to discharge. Final laboratory evaluation demonstrated hemoglobin 10.6 g/dL, white blood cell count 8,810/μL, and platelet count 75-80 × 10³/μL. This patient's laboratory tests are presented in Table 1.

**Table 1:** Serial laboratory findings during the patient's PICU admission Values are presented at PICU admission, the highest (or lowest where clinically relevant) recorded value during hospitalization, and before discharge. Reference ranges may vary slightly between laboratories. PICU: pediatric intensive care unit, PCR: polymerase chain reaction, PaCO₂: arterial partial pressure of carbon dioxide

Parameter	PICU admission	Peak/Nadir during hospitalization	Before discharge	Reference range
Hemoglobin (g/dL)	7.5	6.8	10.6	11.5-15.5
White blood cell count (×10⁹/L)	12.8	14.2	8.81	4.5-13.5
Neutrophils (%)	78	82	55	40-70
Lymphocytes (%)	19	12	35	20-45
Platelet count (×10⁹/L)	45	38	75-80	150-450
C-reactive protein (mg/L)	170	170	9.6	<5
Procalcitonin (ng/mL)	13.3	18.3	0.52	<0.5
Sodium (mmol/L)	125	123	136	135-145
Albumin (g/dL)	2.6	2.4	3.8	3.5-5.0
Aspartate aminotransferase (U/L)	42	58	31	10-40
Alanine aminotransferase (U/L)	28	35	24	7-45
D-dimer (µg/mL)*	3.8	5.1	0.9	<0.5
Ferritin (ng/mL)	650	920	210	20-300
Arterial pH	7.09	7.05	7.4	7.35-7.45
PaCO₂ (mmHg)	70	74	41	35-45
Lactate (mmol/L)	4.2	5	1.3	0.5-2.0
rK39 antigen test	Positive	-	-	Negative
Anti-*Leishmania* antibody titre	Elevated	-	-	Negative
Measles PCR (nasopharyngeal swab)	Positive	-	-	Negative

**Figure 2:**
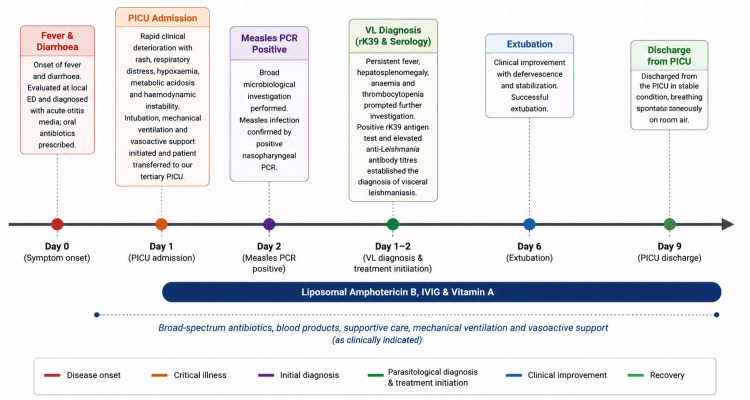
Timeline of the patient's clinical course and therapeutic interventions ED: emergency department, PICU: pediatric intensive care unit, PCR: polymerase chain reaction, rK39: recombinant kinesin antigen test, VL: visceral leishmaniasis, IVIG: intravenous immunoglobulin, Vit A: vitamin A Image Credit: Authors using BioRender.com (BioRender, Ontario, Canada)

The patient improved gradually with normalization of laboratory parameters and hemodynamic stability. He was discharged from the PICU after nine days. Follow-up at four weeks demonstrated full recovery without residual sequelae. The timeline of all these interventions for this toddler is shown in Figure 2.

## Discussion

Measles, caused by the single-stranded RNA virus of the Paramyxoviridae family, is among the most contagious infectious diseases globally, with a basic reproduction number (R₀) estimated at 12-18 [6]. Transmission occurs primarily through respiratory droplets and aerosolized particles, and infection can lead to severe complications, including pneumonia, encephalitis, and profound immunosuppression [1]. Despite the widespread availability of vaccines, gaps in immunization continue to lead to substantial morbidity and mortality, as reported by the WHO [2].

Measles-induced immunosuppression is a well-recognized, multifactorial entity that follows active infection and may persist for weeks to months, increasing susceptibility to severe secondary infections. This type of immunosuppression is both quantitative and qualitative, affecting innate and adaptive immunity [4]. An additional question raised by this case is whether VL represented a newly acquired infection or a reactivation/progression of a previously subclinical *Leishmania* infection following measles-induced immunosuppression. Although this distinction cannot be established with certainty, the patient's household history did not reveal recent travel outside Greece, where *Leishmania* infantum is endemic. It is therefore plausible that measles-associated immune dysfunction either facilitated progression of a recently acquired asymptomatic infection or impaired immune control of a latent infection. The profound depletion of cellular immunity following measles provides a biologically plausible mechanism for either scenario.

Measles virus primarily infects immune cells that express CD150 (also called SLAM). CD150 is found on activated T cells, B cells, dendritic cells, and macrophages. Measles virus uses CD150 as its main entry receptor in the immune system, thereby driving lymphotropism and immunosuppression. This is a key reason measles causes profound immune suppression (“immune amnesia”), leaving people vulnerable to other infections for weeks to months after recovery. Later in infection, measles can also use nectin-4 to infect epithelial cells in the respiratory tract, thereby promoting viral shedding and transmission, but CD150 is central to immune-cell infection [10-17].

Key mechanisms include depletion of pre-existing memory B and T lymphocytes, impaired dendritic cell function and antigen presentation, and altered cytokine production and profile favoring immune tolerance (increased IL-10 and decreased IFN-γ and IL-12) [10], as well as “immune amnesia,” with loss of immune memory against previously encountered pathogens. Studies have demonstrated that measles can erase 40-70% of pre-existing antibody repertoires [11-14], cause widespread lymphocyte apoptosis, and impair antigen-presenting cell function. Furthermore, measles infection promotes a shift from Th1- to Th2-predominant immune responses, characterized by reduced production of IFN-γ and increased levels of anti-inflammatory cytokines such as interleukin-10 (IL-10). Because intracellular killing of Leishmania parasites depends largely on IFN-γ-mediated macrophage activation, this immunological shift may impair parasite clearance and facilitate the persistence or progression of VL. This mechanism provides a biologically plausible explanation for the coexistence of these two infections in our patient.

This immunosuppression can last weeks to months, increasing susceptibility to secondary infections, which contribute significantly to measles-associated morbidity and mortality [3,4,11,12]. Large population studies have shown increased childhood mortality from non-measles infections for up to three years following measles [4], underscoring the long-term consequences of infection. While secondary bacterial infections, such as pneumonia, gastroenteritis, and otitis media, are well documented, secondary viral infections, such as RSV, are less frequent, and parasitic infections (such as malaria and VL) are extremely rare [11,14]. Interestingly, our patient initially presented with neutrophilic leukocytosis and markedly elevated procalcitonin concentrations, findings atypical for both uncomplicated measles and VL, in which leukopenia is more common. These abnormalities most likely reflected a severe systemic inflammatory response syndrome associated with septic shock and acute respiratory failure. At the same time, concomitant bacterial superinfection could not initially be excluded, thereby justifying the empirical administration of broad-spectrum antimicrobial therapy.

**Figure 3:**
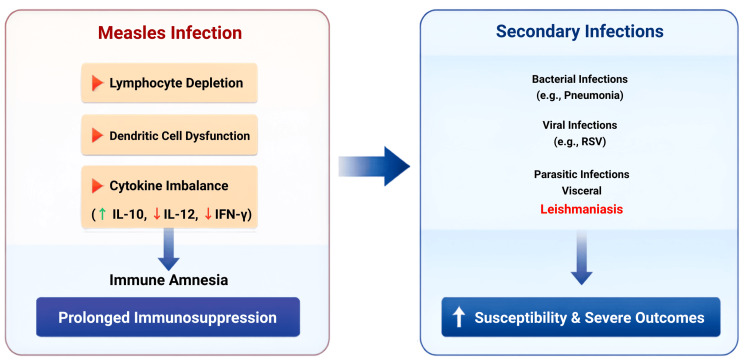
Mechanisms of measles-induced immunosuppression and increased susceptibility to secondary infections IL: interleukin, IFN-γ: interferon-γ, RSV: respiratory syncytial virus Image Credit: Authors using BioRender.com (BioRender, Ontario, Canada)

A schematic representation of the immune dysfunction described is shown in the image below (Figure 3).

VL is a severe systemic parasitic infection caused by *Leishmania donovani* complex protozoa, transmitted by phlebotomine sandflies. VL is endemic in parts of Europe, Africa, the Middle East, and Asia, with children being particularly vulnerable due to immature immune systems [7]. Classic clinical features include prolonged fever, weight loss, hepatosplenomegaly, pancytopenia, and, if untreated, mortality rates exceeding 90% [7,13]. Pediatric patients often exhibit atypical manifestations, making early diagnosis challenging. Diagnosis relies on serology (e.g., rK39 antigen), PCR, or tissue aspiration [8]. VL, although rare, should be considered in children presenting with prolonged fever, hepatosplenomegaly, cytopenias, and septic shock, particularly in endemic regions. These classical features could overlap substantially with severe viral infections and may delay diagnosis. Examples of overlapping clinical features are summarized in Table 2. In regions with even low endemicity of leishmaniasis, clinicians should maintain a high index of suspicion when measles patients present with atypical or prolonged courses. The differential diagnosis in this patient also included secondary HLH, given the presence of persistent fever, hepatosplenomegaly, cytopenias, and hyperferritinemia. However, the patient did not meet accepted diagnostic criteria for HLH, and the rapid clinical improvement following initiation of liposomal amphotericin B, together with normalization of inflammatory markers, supported a diagnosis of VL as the principal underlying condition.

**Table 2:** Co-infection challenges: overlapping clinical features of measles and VL VL: visceral leishmaniasis

Feature	Measles	VL	Co-infection implication
Fever	Acute	Prolonged	May mask VL
Rash	Maculopapular	Rare	Aids measles diagnosis
Hepatosplenomegaly	Mild	Prominent	Early VL may be missed
Cytopenias	Mild	Severe	May persist post-measles
Immunosuppression	Profound	Immunomodulatory	Increased VL severity

Management requires a multidisciplinary approach that integrates intensive care support, targeted antiparasitic therapy, and immunomodulatory interventions, such as vitamin A and intravenous immunoglobulin. Liposomal amphotericin B remains the treatment of choice for pediatric VL due to its favorable efficacy and safety profile [9]. Enhanced infectious disease surveillance is needed to report cases of unusual co-infections, inform clinical guidelines, and implement new public health strategies.

In this case, the coexistence of measles and VL likely contributed synergistically to the severity of illness, leading to septic shock, respiratory failure, and multiorgan involvement. It also underscores the importance of maintaining a high index of suspicion for atypical co-infections in critically ill pediatric patients with measles. It highlights the value of comprehensive microbiological investigation. Secondary parasitic infections following measles are rarely reported, with only sporadic cases in adult populations (e.g., malaria) but no documented pediatric cases to date. Published reports of parasitic co-infections associated with measles are extremely limited and appear to consist mainly of isolated cases or small case series. Although bacterial secondary infections are well recognized following measles, pediatric measles-associated VL has not, to the best of our knowledge, been previously documented. Control of *Leishmania* infection relies heavily on cell-mediated immunity. Children with impaired Th1 responses are therefore particularly vulnerable to VL [8,9]. Although parasitological confirmation by bone marrow examination or molecular testing was not performed, the diagnosis was supported by the characteristic clinical presentation, positive rK39 antigen testing, elevated anti-*Leishmania* antibody titers, and the patient's prompt clinical response to liposomal amphotericin B. Nevertheless, the absence of direct parasitological confirmation should be acknowledged as a limitation of this report.

From an epidemiological perspective, Greece remains endemic for *Leishmania infantum*, while periodic measles outbreaks continue to occur despite the availability of effective vaccination. Although both infections may occur independently within the same geographical region, their coexistence has not previously been reported in the pediatric population, emphasizing the exceptional nature of this case and the importance of maintaining a broad differential diagnosis in critically ill children with persistent fever, hepatosplenomegaly, and cytopenias. Early identification of both pathogens and prompt initiation of pathogen-directed therapy were critical to this patient's favorable outcome. This particular case also highlights the far-reaching consequences of inadequate vaccination coverage. Measles vaccination not only prevents the acute viral illness but also protects against its complications and prevents secondary infections mediated by immune amnesia. In endemic regions, preventing measles may indirectly reduce the incidence and severity of diseases such as VL. Strengthening public health strategies, such as immunization programs, improving surveillance, and enhancing clinicians' awareness of rare complications, remain essential to reducing pediatric morbidity and mortality.

We report the first pediatric case of measles-VL co-infection, highlighting the profound immunosuppressive effects of severe measles infection. The child developed a life-threatening clinical course complicated by shock, diffuse viral pneumonitis with respiratory failure, and marked hepatosplenomegaly. During the post-measles immunosuppressive period, further investigation led to the diagnosis of VL. Prompt initiation of liposomal amphotericin B therapy resulted in rapid clinical improvement, normalization of cardiac function, and complete recovery. This case underscores the importance of considering opportunistic and endemic infections in children with severe measles. It emphasizes the critical role of vaccination in preventing life-threatening secondary infections and complications.

## Conclusions

To the best of our knowledge, no pediatric case of measles-VL co-infection has been documented previously. Although secondary parasitic infections following measles are exceptionally rare, this case demonstrates that VL should be considered in children with persistent fever, hepatosplenomegaly, and cytopenias despite appropriate supportive management, particularly in endemic regions.

This case highlights the profound immunosuppressive effects of measles and emphasizes the need for heightened clinical vigilance and early recognition of atypical secondary infections. It also highlights the importance of early diagnostic evaluation in children with persistent fever and cytopenias, as well as the critical role of vaccination in preventing life-threatening complications.

Prompt recognition and a multidisciplinary therapeutic approach were pivotal in achieving a favorable outcome. Measles vaccination remains the most effective strategy for preventing severe disease, measles-associated immune dysfunction, and its potentially life-threatening secondary infectious complications. Clinicians should maintain a broad differential diagnosis, including VL, in children with severe measles who present with persistent fever, progressive hepatosplenomegaly, and cytopenias, particularly in endemic or partially endemic regions.
